# Supplementary material for: Multimodal imaging and functional analysis of the chick NMDA retinal damage model
Source: PLoS One. 2021 Sep 7;16(9):e0257148. doi: 10.1371/journal.pone.0257148 (PMC8423281; doi:10.1371/journal.pone.0257148)
Supplement: S1 File — (ZIP) [file pone.0257148.s004.zip › SD-OCT/C200727/006/D15-PWk3/C200727-006_OD_R_14_0_REGAVG0000100/Manual_PreDefineThreeByFourGridRadialRetina_1.pdf]

# Three By Four Grid Radial Retina Template Report

| | | | |
|---|---|---|---|
| **Patient ID** | C200727 | **Name** | See InVivoVue |
| **Exam Date** | See InVivoVue | **Exam Time** | See InVivoVue |
| **Eye** | OD | **Averaged** | N/A |
| **Depth (mm)** | 1.65 | **Depth Samples** | 1024 |
| **Pitch (um/pix)** | 14.000 x 2333.333 x 1.608 | **Refractive Index** | See InVivoVue |
| **Scan Type** | Radial 14.000mm x 14.000mm x 1.647mm ,<br>1000 x 6 x 1024 | | |

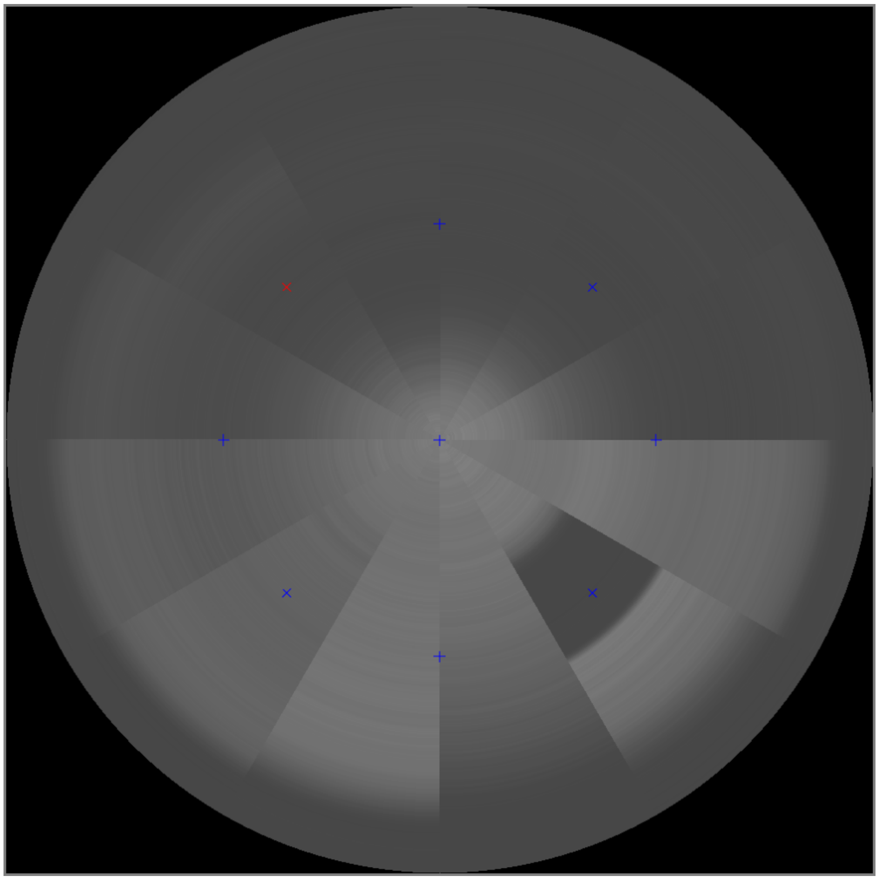

## Thickness Metrics

| Analysis | Average(mm) | Minimum(mm) | Maximum(mm) | STDEV(mm) |
|---|---|---|---|---|
| **IRNFL - RPE** | 0.2678 | 0.2446 | 0.3021 | 0.0206 |
| **IRNFL - ORNFL** | 0.0437 | 0.0312 | 0.0630 | 0.0125 |
| **ORNFL - OPL** | 0.1450 | 0.1173 | 0.1585 | 0.0129 |
| **OPL - ETPRS** | 0.0594 | 0.0537 | 0.0668 | 0.0045 |
